# Applying North American medical education accreditation standards internationally in the United Arab Emirates

**DOI:** 10.1080/10872981.2022.2057790

**Published:** 2022-03-27

**Authors:** Sandra Kay Allen, Zahra S. Baalawi, Ahmed Al Shoaibi, Hesham Wagih Gomma, John A. Rock

**Affiliations:** a College of Medicine and Health Sciences, Khalifa University, Abu Dhabi, UAE; b Academic and Student Services, Khalifa University, Abu Dhabi, UAE; c Institutional Research and Planning, Khalifa University, Abu Dhab

**Keywords:** Medical education, program evaluation, international medical education accreditation, LCME accreditation, continuous quality improvement

## Abstract

**Objective:**

Health care and health professions education are becoming increasingly global, yet no formal international accrediting body exists for medical education. Among the challenges in developing international standards for medical education is the variation in program models, with some regions offering six-year bachelor’s degrees and others, including North America, customarily requiring a bachelor’s degree prior to admission to a 4-year graduate-level degree program. This study sought to determine the applicability of the USA Liaison Committee on Medical Education (LCME) accreditation standards internationally as the foundation for program development, quality improvement, and program evaluation in a program that follows the North American medical education model in the United Arab Emirates (UAE).

**Methods:**

Using a qualitative political, economic, sociocultural, technological, legal, and environmental (PESTLE) analysis framework, we systematically assessed the applicability of each of the 93 LCME accreditation elements to the nascent doctor of medicine (MD) degree program at Khalifa University.

**Results:**

All 93 elements in the most current LCME accreditation standards were deemed applicable internationally in a program developed in accordance with the North American model of medical education. Of these, three elements were deemed applicable with caveats in the legal or regulatory processes required to achieve comparable compliance outside of the USA. No elements were deemed not applicable in an international setting.

**Conclusions:**

Our analysis demonstrates that the LCME accreditation standards are model-specific and can be effectively applied internationally in programs that follow the North American model of medical education. Countries in which no specialized medical education accrediting body exists can apply the LCME standards and achieve international benchmarks of quality in medical education through rigorous self-assessment and continuous quality improvement.

## Introduction

Over the past several decades, healthcare and health professions education have become increasingly global as practitioners, patients, and students cross national borders for jobs, better or cheaper healthcare, and educational opportunities. Although somewhat hindered by the Covid-19 pandemic in 2020–2021, medical education also is becoming more global, with increasing numbers of medical schools establishing branch campuses in other countries and students seeking admission abroad [1–6]. In 2019, forty-eight USA medical schools accredited by the Liaison Committee on Medical Education (LCME), the formal accrediting authority for allopathic medical education programs in the USA, indicated that they accept applications from international students[7]. In addition, the majority of USA medical schools offer opportunities for students to take electives abroad and many allow international visiting students to take electives at their institutions[8]. Despite these trends, accreditation of medical education programs continues to occur at the national level, with global standards provided as guidance to national accrediting bodies but with no formal process for international accreditation [9,10]. Among the challenges in developing international standards for medical education is the variation in program models across different regions of the world. Many regions offer six-year bachelor’s degrees, while other regions, including North America, customarily require a bachelor’s degree prior to admission to a 4-year graduate-level Doctor of Medicine (MD) degree program. Some North American medical schools offer accelerated, fast-track, or combined- or dual-degree programs that enable students to earn both a bachelor’s degree and a graduate level MD degree in as few as 6 or 7 years[11].

### Medical education in the United Arab Emirates

The United Arab Emirates (UAE) has made tremendous strides in medical education since the establishment of its first federal medical school in the mid-1980s. Currently, eight medical schools are accredited by the Commission for Academic Accreditation (CAA), the federal government agency responsible for the accreditation of all higher education institutions and academic programs in the UAE [12–14]. Seven UAE medical schools offer Bachelor of Medicine/Bachelor of Surgery (MBBS) degree programs for which a high school diploma is required before entry to a program that is typically 6 years’ duration (Table 1). When planning the nation’s newest medical school at Khalifa University in Abu Dhabi, Khalifa University sought to develop an MD degree program that is compliant with the CAA standards and is contextualized to the UAE’s health priorities, but follows the North American model of medical education awarding a graduate-level MD degree [15,16]. Although the LCME does not accredit programs outside of the USA, we sought to demonstrate that the LCME standards are designed for the model of education rather than the country in which they are applied, and thus can be applied as the foundation for program development, quality improvement, and program evaluation at Khalifa University or other programs anywhere in the world that follow a similar model.

**Table 1.** Medical schools in the United Arab Emirates in 2021

Medical Education Programs by Emirate	Established	Degree	Duration	QFEmirates Level
Abu Dhabi				
Khalifa University	2019	MD	4 Years	9
United Arab Emirates University	1986/2012	MBBS/MD	6 Years	7
Ajman				
Ajman University	2018	MBBS	6 years	7
Gulf Medical University	1998	MBBS	6 Years	7
Dubai				
Dubai Medical College for Girls	1986	MBBS	6 Years	7
Mohammed Bin Rashid University of Medicine and Health Sciences	2016	MBBS	6 Years	7
Ras Al Khaimah				
Ras Al Khaimah Medical and Health Sciences University	2006	MBBS	6 Years	7
Sharjah				
University of Sharjah	2004	MBBS	6 Years	7

MD indicates Doctor of Medicine; MBBS, Bachelor of Medicine, Bachelor of Surgery; QF*Emirates* is UAE’s National Qualifications Framework for levels of higher education degrees.

Sources: Commission for Academic Accreditation (CAA) [12]; World Federation for Medical Education (WFME) [13].

## Methods

### Design

A qualitative design similar to that reported by Walsh et al [17] was used to assess the impact of political, economic, sociocultural, technological, legal, and environmental (PESTLE) forces on the application of the LCME standards to the Khalifa University MD degree program in the UAE[18].

### Data collection

We gathered empirical evidence on the extent to which the six PESTLE external forces or conditions impact the application of each of the 93 LCME elements[15]. Two authors (SKA, JAR) with previous experience with LCME accreditation independently assessed the applicability of each LCME element in the UAE. Elements were deemed ‘applicable’ if it is possible for a program following the North American model of undergraduate medical education in the UAE to comply with the LCME standards. Elements were deemed ‘applicable with caveat’ when one or more PESTLE factors impact the mechanism or processes for complying with the LCME standards. Elements were deemed ‘not applicable’ if we determined that it is not possible to achieve comparable compliance with the LCME standards in the UAE. Inter-rater reliability was high and elements for which initial assessments differed were jointly discussed by all authors until a consensus was reached. One author (HWG) with extensive experience in UAE accreditation processes provided expertise concerning the comparability of the LCME standards with those of the UAE’s national higher education accrediting body. Two authors (ZSB, AAS) are UAE Nationals (citizens) and were instrumental in assessing the impact of PESTLE forces on compliance from the national perspective as summarized below.

### Political forces

The UAE is a politically stable constitutional federation of seven Emirates, each of which has its own ruler and government structure. Khalifa University is an independent, non-profit, coeducational institution funded by and accountable to the government of the Emirate of Abu Dhabi.

### Economic forces

The UAE is economically stable, with low unemployment rates and a high gross domestic product. The nation’s strategic vision, and the strategic vision of the Emirate of Abu Dhabi, are focused on diversifying the economy by reducing reliance on oil and increasing knowledge-based industries, including education and health care.

### Sociocultural forces

Census data indicate that the UAE currently has a total population of approximately 9.89 million, of which approximately 12% are UAE Nationals. The nation relies heavily on its expatriate workforce and expatriates benefit from the nation’s significant investments in the health and safety of its people. The Khalifa University MD degree program supports the UAE’s Emiratization initiative, which aims to increase the number of Emiratis in the workforce, including in health professions. English is the official language of instruction in most higher education institutions in the UAE, including Khalifa University. Islam is the official religion in the UAE and because Islamic holidays are determined according to the sighting of the moon, medical schools require flexibility in establishing academic calendars and scheduling proctored examinations. Khalifa University maintains a conservative atmosphere but its academic programs are co-educational.

### Technological forces

The UAE invests heavily in research and development of advanced technology and is highly ranked in world technology indices. Khalifa University invests heavily in state-of-the-art equipment and technologies, which are readily available to support its academic and research programs.

### Legal forces

The constitution of the UAE outlines the freedoms, rights, and responsibilities of the nation’s citizens and residents. Government authorities oversee the various sectors of the economy and public life. The UAE Commission for Academic Accreditation (CAA) is the federal government authority for licensure and accreditation of higher education institutions nationwide.

### Environmental forces

The UAE is geographically located in the north-eastern Arabian Peninsula, bordering with Oman to the north and east, Saudi Arabia to the south, the Arabian Gulf to the north and west, and the Gulf of Oman to the east. These coastal borders facilitate easy trade by sea, which is essential because the desert climate and weather patterns present challenges such as desertification and freshwater scarcity, requiring the nation to import the majority of its food.

## Results

All 93 elements in the most current version of the LCME accreditation standards were deemed applicable in the UAE as an effective method of medical education program evaluation. Of these, three elements required caveats to meet the LCME accreditation criteria in the UAE. In our analysis of external PESTLE forces, all three caveats involved legal forces impacting regulatory processes as described below (Table 2). No negative impacts were noted from political, economic, sociocultural, technological, or environmental forces. No elements were deemed not applicable in an international setting.

**Table 2.** PESTLE analysis: legal forces impacting applicability of LCME elements in the UAE

LCME Element	LCME Requirement	Caveat
1.6 Eligibility Requirements	Parent institution accredited by a USA regional accrediting body	Parent institution accredited by a UAE national accrediting body
5.12 Required Notifications to the LCME	Substantial changes impacting resources must be reported in advance to LCME	Substantial changes impacting resources must be reported in advance to CAA
8.4 Evaluation of Educational Program Outcomes	Reported outcome measures include results from USA residency match program, licensing examinations, and national student survey	Reported outcome measures include results from UAE residency match program, licensing examinations; national student survey to be developed

### Caveat 1: LCME Element 1.6 eligibility requirements

To be eligible for LCME accreditation, a medical school or its parent institution must be accredited by one of the six USA regional accrediting organizations[15]. In the UAE, a comparable process exists at the national level for accreditation and licensing of all higher education programs by the CAA[16].

### Caveat 2: LCME Element 5.12 required notifications to the LCME

Medical schools accredited by the LCME are expected to notify the LCME in advance of substantive changes that impact the resources of the medical education program. For example, the LCME accreditation process evaluates a school’s ability to support a specified number of students per cohort. Any increase of greater than 10% or 15 students (whichever is smaller) must be reported so that the LCME can evaluate the school’s ability to support the increase[15]. In the UAE, the CAA has a comparable requirement for notification of substantive changes, requiring institutions to ensure that class sizes are consistent with international best practices and that the student/faculty ratio is appropriate for the pedagogy applied and the level of the course[16].

### Caveat 3: LCME Element 8.4 evaluation of educational program outcomes

The LCME requires schools to report USA national norms, including student match rates in the National Residency Matching Program (NRMP), student results on national licensing examinations, and graduate survey data derived from the Association of American Medical Colleges (AAMC) Medical School Graduation Questionnaire[15]. In the UAE, different national norms apply. It is anticipated that some but not all medical students at Khalifa University may apply for residency training in North America, therefore Khalifa University will evaluate match rates in residency programs worldwide, including the USA, Canada, and the UAE Department of Health TANSEEQ residency match. It is also anticipated that some Khalifa University medical students will take the USMLE Step examinations for qualification for USA residency training; however, the International Foundations of Medicine (IFOM) examinations administered by the USA National Board of Medical Examiners currently serves as the national norm in the UAE.

## Discussion

The development of the PESTLE analysis framework evolved from the work of Francis Aguilar, a business professor who first introduced the approach as a means for understanding how external forces impact a business organization[18]. Our PESTLE analysis identified three LCME accreditation elements for which legal forces, including the regulatory requirements of accrediting agencies, impact the mechanisms or processes required to apply the accreditation criteria in the UAE. The implication of these caveats is that international application is possible using alternate but comparable measures of compliance within the country’s legal and regulatory frameworks.

### Advocates for international accreditation: WFME and WHO

Since 2005, the World Federation for Medical Education (WFME) has maintained nonbinding global standards for basic medical education across the continuum from undergraduate to graduate to continuing medical education[10]. The WFME global standards provide guidance to medical education programs worldwide, enabling them to voluntarily implement and self-assess quality improvement processes. The WFME does not conduct formal accreditation processes or confer accreditation status to medical schools, rather it evaluates and confers Recognition Status to national accrediting agencies whose processes meet internationally accepted high standards of quality. Medical schools accredited by agencies recognized by the WFME are eligible to be listed in the World Directory of Medical Schools, and qualified graduates of those schools are eligible to seek certification from the USA Educational Commission for Foreign Medical Graduates (ECFMG)[13]. This is of critical importance for foreign medical graduates seeking medical residency training positions in the USA.

In 2013, the World Health Organization (WHO) advocated for the development of ‘an internationally sanctioned system’ of accreditation of health professional programs[19]. The WHO also encouraged countries with general higher education accreditation systems to develop related systems of accreditation specifically for health professions education[19]. More recently in 2016, the WHO set a goal for all countries to establish accreditation mechanisms for health training institutions by 2020[20]. In the UAE, the CAA released updated standards in 2019, before the WHO’s 2020 target date. The CAA’s 2019 standards include broad criteria for the appointment of clinical faculty and physical and organizational requirements of teaching hospitals and healthcare units within the UAE, but do not include content-specific criteria typically included in specialized accreditation standards for medical education and other disciplines[16].

### Using the LCME Standards for self-assessment and continuous quality improvement

The LCME requires new and developing medical schools to undergo three separate LCME cycles (before enrolling students, before starting the clinical phase, and before graduation of the first cohort) within approximately five years. Each cycle involves data collection, self-study, and peer review. After full accreditation is conferred by the LCME, reaccreditation occurs every eight years – a timeframe within the four- to ten-year range used by the majority of medical school accreditation bodies worldwide[21]. In the UAE, the CAA follows a similar incremental accreditation process with reaccreditation cycles stratified from 3 to 7 years using a risk-based-approach[16]. The most current LCME accreditation data collection instrument is structured with twelve broad-category standards, each with six to twelve detailed elements (a total of 93 elements), and includes numerous tables for aggregating data from various sources[15]. The standards are updated frequently and are available in the public domain via lcme.org. The LCME self-study process engages members of the medical education community in identifying program vulnerabilities. A well-executed self-study often leads to timely quality improvement interventions that preempt adverse findings and severe action decisions by the LCME [21–23]. Recognizing that eight-year review cycles may be too long to ensure continued compliance, in 2015 the LCME introduced a formal component (Element 1.1) requiring schools to ‘ensure effective monitoring of the medical education program’s compliance with accreditation standards,’ thus formalizing the requirement for continuous quality improvement (CQI) processes[24]. Similarly, the CAA also requires multiple cycles of assessment by external review teams, with standards for quality assurance and quality improvement that are broadly applicable to all disciplines of higher education[16].

Our work dispels a misconception that the LCME standards are applicable only in the USA. Indeed, our results demonstrate that, even in countries in which no independent, specialized medical education accrediting body exists, medical schools following the model on which the LCME standards are based (i.e., graduate-level professional MD degree program) can voluntarily apply the LCME standards and, thus, raise the bar in achieving international best practices through self-evaluation and continuous quality improvement[25].

### Study limitations

Khalifa University’s use of the North American graduate-degree model is an important distinction that differentiates its academic program from the other UAE medical schools offering baccalaureate degrees in medicine or surgery. The results of our analysis of the applicability of the LCME standards at Khalifa University cannot be generalized to programs that do not follow the LCME-sanctioned model. For example, standards related to admissions criteria and curricular content vary significantly between six-year bachelor’s degree programs and graduate-level professional MD degree programs.

## Conclusions

The quality of medical education is a priority for all nations striving to develop quality healthcare systems. Although no formal international medical education accrediting agency currently exists, national accrediting agencies apply various approaches to ensure that medical education programs meet defined standards of quality. Our analysis demonstrates that the LCME accreditation standards can be voluntarily and effectively applied internationally in programs that follow the North American model and conduct robust self-assessment and continuous quality improvement to achieve international benchmarks of quality in medical education.
